# SEVERE ALZHEIMER’S DEMENTIA ALTERS THE RELATIONSHIP BETWEEN EXECUTIVE FUNCTION AND FALLS

**DOI:** 10.1093/geroni/igad104.3588

**Published:** 2023-12-21

**Authors:** Ferdinand Delgado, Fang Yu, David MacKinnon, Daniel Peterson, Edward Ofori, Thomas Beach, Charles Adler, Cheryl Der Ananian

**Affiliations:** University of New Hampshire, Durham, New Hampshire, United States; Arizona State University, Phoenix, Arizona, United States; Arizona State University, Tempe, Arizona, United States; Arizona State University, Phoenix, Arizona, United States; Arizona State University, Tempe, Arizona, United States; Banner Sun Health Research Institute, Sun City, Arizona, United States; Mayo Clinic, Scottsdale, Arizona, United States; Arizona State University, Phoenix, Arizona, United States

## Abstract

Falls are a public health concern for older adults, particularly those with cognitive impairments, like mild cognitive impairment (MCI) and Alzheimer’s disease (AD) dementia. Executive function (EF) is linked to falls in older adults, but little is known about the relationship between individual EF assessments and falls in individuals with varying cognitive statuses. Using cross-sectional secondary data analyses with binary logistic regression, we examined the associations between EF assessments (Trail Making Test (ΔTMT), Digit Span (DS), Stroop test, Clock Drawing Test, and phonemic/semantic fluency tests) and falls (fall history and subsequent falls) in 466 participants (aged 66-103) with either intact cognition, MCI, or AD dementia. Surprisingly, a significant interaction effect emerged with AD dementia and EF assessments (ΔTMT, DS, and semantic fluency), revealing that individuals with AD dementia with worse EF had a lower probability of prior fall history. These findings were influenced by a low percentage of reported falls in people with severe AD dementia. Further stratified analysis of the interaction effects showed that DS and semantic fluency were significantly associated with fall history in people without AD dementia. While some EF assessments were individually associated with falls, interpretations should be made cautiously, especially considering the appropriateness of the assessments for specific populations. The counterintuitive result may stem from factors specific to severe AD dementia. In conclusion, this study uncovers complex interactions between cognitive status, EF assessments, and fall history, and the potential for further investigation to enhance our understanding of falls in individuals with severe AD dementia.

